#  Rustrela Virus in Wild Mountain Lion (*Puma concolor*) with Staggering Disease, Colorado, USA

**DOI:** 10.3201/eid3008.240411

**Published:** 2024-08

**Authors:** Karen A. Fox, Angele Breithaupt, Martin Beer, Dennis Rubbenstroth, Florian Pfaff

**Affiliations:** Colorado State University College of Veterinary Medicine and Biomedical Sciences, Fort Collins, Colorado, USA (K.A. Fox);; Colorado Parks and Wildlife, Fort Collins (K.A. Fox);; Friedrich-Loeffler-Institut, Greifswald-Insel Riems, Germany (A. Breithaupt);; Institute of Diagnostic Virology, Friedrich-Loeffler-Institut, Greifswald-Insel Riems (M. Beer, D. Rubbenstroth, F. Pfaff)

**Keywords:** Rustela virus, rubivirus, viruses, encephalomyelitis, encephalitis, ataxia, paresis, puma, mountain lion, cats, staggering disease, Colorado, United States

## Abstract

We identified a rustrela virus variant in a wild mountain lion (*Puma concolor*) in Colorado, USA. The animal had clinical signs and histologic lesions compatible with staggering disease. Considering its wide host range in Europe, rustrela virus should be considered as a cause for neurologic diseases among mammal species in North America.

On May 12, 2023, Colorado Parks and Wildlife (Denver, CO, USA) received a report of an ≈1-year-old free-ranging female mountain lion (*Puma concolor*) with signs of severe hind leg ataxia and paresis. The lion had been observed in a residential area of Douglas County, Colorado, USA (Appendix Figure 1). The animal was reluctant to rise and had markedly decreased capacity to move or bear weight on the hind end (Video). The animal appeared depressed but was still responsive to stimuli. Wildlife officers tranquilized the animal and then euthanized it by gunshot to the chest to prevent destruction of neurologic tissues. We conducted a postmortem investigation including necropsy, histopathology, immunohistochemistry, molecular diagnostics, and metatranscriptome sequencing to investigate potential causes of the disease.

**Video:** An approximately 1-year-old female mountain lion (*Puma concolor*) with impaired mobility. The mountain lion struggles to rise and staggers forward with difficulty because of hind limb ataxia and paresis. Other features of the animal’s gait include swaying of the hips, slight head tremors, and repeated collapse. The overall mentation of the animal is depressed. Video captured by a homeowner in Douglas County, Colorado, USA, on May 12, 2023.

## The Study

**Figure 1:**
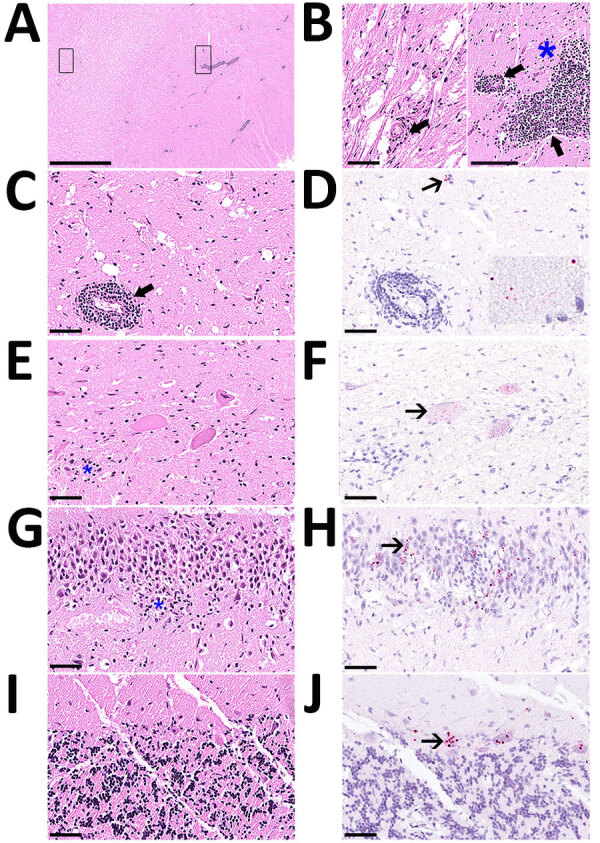
Histology of brain and spinal cord used to detect rustrela virus (RusV) in wild mountain lion (*Puma concolor*) with staggering disease, Colorado, USA. RusV RNA was detected by RNAscope Reagent Kit-Red (Advanced Cell Diagnostics/bio-techne, https://www.bio-techne.com) in situ hybridization**.** All sections demonstrate artifactual clefting of the neuropil due to freezing of the tissue postmortem. A) Cerebral cortex with perivascular cuffing and mild gliosis of the white and gray matter; boxes indicate detailed areas in panel B. Scale bar indicates 2.5 mm. B) The white matter (left panel) is minimally affected by perivascular lymphohistiocytic infiltrates (bold arrow), compared with the gray matter (right panel), also showing gliosis (asterisk). Scale bar indicates 100 µm. C) Midbrain affected by perivascular cuffing. Scale bar indicates 50 µm. D) Midbrain showing chromogenic labeling (fast red) of RusV in neuronal cell bodies (slender arrow) and in the neuropil (inlay). Scale bar indicates 50 µm. E) Spinal cord with 3 motor neurons showing variable degree of degeneration/necrosis and also gliosis (asterisk). Scale bar indicates 50 µm. F) Spinal cord with affected motor neurons with RusV RNA detection. Scale bar indicates 50 µm. G) Hippocampus exhibiting irregular architecture of the granule layer and gliosis (asterisk). Scale bar indicates 50 µm. H) Hippocampus with numerous RusV RNA signals in neurons of the granule cell layer in areas with or without irregular architecture. Scale bar indicates 50 µm. I) Cerebellum, no indication for inflammation or any degenerative process. Scale bar indicates 50 µm. J) Cerebellum with abundant RusV RNA labeling in Purkinje cells. Scale bar indicates 50 µm. A–C, E, G, I) Hematoxylin-eosin staining; D, F, H, I) RNAscope in situ hybridization with probes against the nonstructural protein–coding region of RusV, counterstained with Mayer’s hematoxylin.

Prenecropsy radiology revealed no skeletal abnormalities to explain the clinical signs observed. Necropsy results indicated poor body condition and mild bruising at the torso and limbs. The stomach contained only pine needles. Histopathology demonstrated severe nonsuppurative meningoencephalomyelitis (Appendix). The leptomeninges were multifocally and markedly expanded by lymphocytes and histiocytes in both brain and spinal cord (Figure 1). Virchow-Robin perivascular spaces were expanded by dense cuffs of lymphocytic to lymphohistiocytic infiltrates up to 20 cell layers thick in nearly all regions of the brain (Figure 1, panels A–C) and spinal cord. Inflammation was largely restricted to the leptomeninges and gray matter, and only minimal in the white matter (Figure 1, panels A, B). Affected sections also demonstrated scattered neuronal necrosis, gliosis, and loose glial nodules (Figure 1, panels B, E, G), partially leading to an irregular architecture (Figure 1, panel G). The cerebellar cortex showed no indication of inflammation or degenerative process (Figure 1, panel I)

Initial diagnostic tests did not detect feline panleukopenia virus, canine distemper virus, West Nile virus, *Toxoplasma gondii*, influenza A virus, rabies virus, or feline infectious peritonitis virus in the central nervous system (Appendix Table 1). We used pooled brain and spinal cord tissue to extract total RNA (Appendix), then conducted metatranscriptome sequencing to obtain sequence fragments (reads). We used those fragments to de novo assemble a single contiguous sequence (contig) with homology to known sequences of rustrela virus (RusV). The contig represented the whole viral genome and matched RusV reference strains. We submitted the annotated RusV genome sequence to the International Nucleotide Sequence Database Collaboration (https://www.insdc.org; accession no. PP025855). 

We adapted real-time reverse transcription PCR primers and probe for RusV (*1*) by using degenerate bases for consensus homology to the Colorado mountain lion–derived sequence and European RusV sequences (Appendix Table 2). Those adapted methods showed RusV RNA in a pooled sample of brain and spinal cord from the mountain lion, with a cycle threshold value of 20.3.

RusV (*Rubivirus strelense*), a member of the family *Matonaviridae*, was recently identified as the cause of staggering disease (*1*), a usually fatal neurologic syndrome in cats. Since the 1970s, staggering disease has been documented in domestic cats in Europe, predominantly in Sweden and Austria (*2*–*6*). Affected cats show a consistent combination of histologic lesions and clinical signs, including hind limb ataxia or paresis, and nonsuppurative meningoencephalitis restricted to the gray matter but not affecting white matter or the cerebellar cortex (*1*,*4*,*5*). A similar syndrome was reported in cats from Alabama, USA, in 1979 but the etiology remained obscure (*7*). In Germany, RusV has been detected in a broad range of zoo animals with neurologic disorders, including lions (*Panthera leo*) (*8*–*11*).

Because initial diagnostic tests were negative in this case, and history, histopathology, and metatranscriptome sequencing suggested staggering disease, we sent tissue samples and sequence data from the mountain lion to the Friedrich-Loeffler-Institut (Greifswald-Insel Riems, Germany) for additional analyses. To demonstrate an association between the lesions and the virus, we used previously developed in situ hybridization methods for RusV (*1*,*6*) (Appendix), which demonstrated RusV RNA in all regions of the brain and nearly all levels of the spinal cord, irrespective of an inflammatory reaction. Only lumbosacral nerve roots (cauda equina) tested negative. RusV-specific RNA localized in neuronal cell bodies (Figure 1, panels D, F, H, J), disseminated within the neuropil of the gray matter (Figure 1, panel D, inlay) and, to a lesser extent, in the white matter. We found particularly abundant or large, dot-like signals in the granule cell layer of the hippocampus (Figure 1, panel H), and in Purkinje cells of the cerebellum (Figure 1, panel J), similar to findings from staggering disease cases in cats from Europe (*4*).

**Figure 2:**
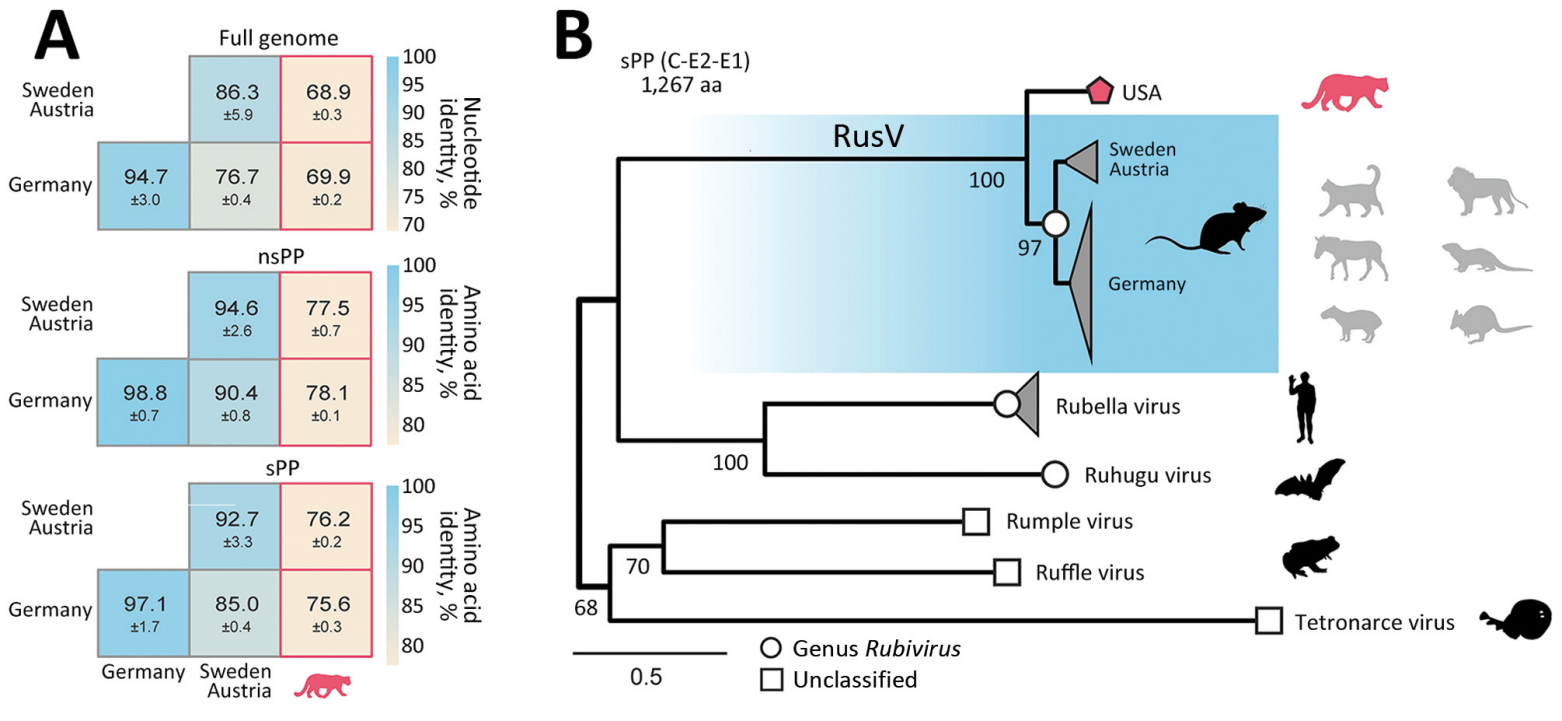
Sequence similarity and phylogenetic position of RusV in wild mountain lion (*Puma concolor*) with staggering disease, Colorado, USA. A) Mean pairwise sequence identity between the novel Colorado RusV and RusV sequences from Germany, Austria, and Sweden. Pairwise identity was based on nucleotide sequence alignments of the full genome or amino acid alignments of the nsPP and sPP. B) The sPP amino acid sequences of appropriate references from rubiviruses (circle) or currently unclassified matonavirids (square) were aligned with the novel RusV (pentagon). Phylogenetic tree was calculated using IQ-TREE (http://www.iqtree.org). Host species are depicted as silhouettes. For RusV, the potential reservoir (dark) and spillover hosts (light) are depicted. Scale bar indicates substitutions per site. nsPP, nonstructural polyprotein; sPP, structural polyprotein.

The overall architecture of the viral genome of the novel RusV from Colorado matched those of known RusV (Appendix Figure 2). The mean pairwise nucleotide identity between the novel RusV sequence and sequences from Germany was 69.9% and between sequences from Austria and Sweden was 68.9%; the sequences from Europe shared 76.7% identity among each other (Figure 2, panel A). The mean pairwise amino acid identities of the nonstructural and structural polyproteins ranged from 75.6% to 78.1% between the novel RusV sequence and the sequences from Europe (Figure 2, panel A). The genetic diversity was not equally distributed over the genome; part of the protease and the intergenic region showed especially high levels of sequence variations (Appendix Figure 2).

We performed phylogenetic analysis to compare the RusV sequence from Colorado with appropriate reference strains using an amino acid alignment of the structural polyprotein (Appendix). Those findings suggested classification of the novel RusV as a member of the family *Matonaviridae*, genus *Rubivirus*, placing it basal to the known RusV sequences detected in Germany, Sweden, and Austria (Figure 2, panel B). The basal position of the novel RusV in relation to all other known RusV is also supported by phylogeny based on the whole-genome nucleotide sequence (Appendix Figure 3). 

## Conclusions

Our results demonstrate the presence of a RusV variant in North America that is divergent from those previously described from Europe. The clinical signs, histologic lesions, and infected target cells observed for the wild mountain lion in Colorado, USA, meet the case definition for staggering disease. A causative role for RusV is likely, further supporting previous work identifying RusV as the causative agent of staggering disease in domestic cats from Austria, Sweden, and Germany (*1*,*6*), and in lions from zoologic collections in Germany (*10*).

This report is limited to a single case of staggering disease in Colorado. To determine whether RusV is enzootic in this region, we recommend further investigations, including retrospective RusV testing of tissues from feline encephalitis cases of unknown causes in North America. Surveillance for RusV in small rodents might identify a local reservoir host because rodents of the genus *Apodemus* have been identified as likely RusV reservoir hosts in Europe through real-time reverse transcription PCR and sequencing of mice brain tissues (*1*,*8*,*9*,*12*,*13*). Although *Apodemus* mice are not indigenous to North America, several genera of small rodents are found throughout Colorado (*14*,*15*) and could serve as candidates for further screenings. In addition, future studies should consider that the zoonotic potential of RusV has not been determined.

Of note, a remarkably broad range of other mammalian RusV hosts has been identified in Germany, including equids, mustelids, rodents, and marsupials (*8*,*9*,*11*,*12*), raising concerns about a zoonotic potential of RusV (*8*,*9*). Given the wide host range of the virus in Europe, RusV should be considered as a possible cause for neurologic diseases in all mammal species in North America. 

Appendix Additional information on detection of rustrela virus in wild mountain lion (*Puma concolor*) with staggering disease, Colorado, USA.

## References

[1] Matiasek K, Pfaff F, Weissenböck H, Wylezich C, Kolodziejek J, Tengstrand S, et al. Mystery of fatal ‘staggering disease’ unravelled: novel rustrela virus causes severe meningoencephalomyelitis in domestic cats. Nat Commun. 2023;14:624. 10.1038/s41467-023-36204-w 36739288 PMC9899117

[2] Lundgren A-L. Feline non-suppurative meningoencephalomyelitis. A clinical and pathological study. J Comp Pathol. 1992;107:411–25. 10.1016/0021-9975(92)90015-M 1291589 PMC7130315

[3] Kronevi T, Nordström M, Moreno W, Nilsson PO. Feline ataxia due to nonsuppurative meningoencephalomyelitis of unknown aetiology. Nord Vet Med. 1974;26:720–5. 4449727

[4] Weissenböck H, Nowotny N, Zoher J. Feline meningoencephalomyelitis (“staggering disease”) [in Austrian]. Wien Tierarztl Monatsschr. 1994;81:195–201.

[5] Nowotny N, Weissenböck H. Description of feline nonsuppurative meningoencephalomyelitis (“staggering disease”) and studies of its etiology. J Clin Microbiol. 1995;33:1668–9. 10.1128/jcm.33.6.1668-1669.1995 7650212 PMC228243

[6] Weiss V, Weidinger P, Matt J, Weissenbacher-Lang C, Nowotny N, Weissenböck H. Rustrela virus-associated encephalomyelitis (‘staggering disease’) in cats from eastern Austria, 1994–2016. Viruses. 2023;15:1621. 10.3390/v15081621 37631964 PMC10458416

[7] Vandevelde M, Braund KG. Polioencephalomyelitis in cats. Vet Pathol. 1979;16:420–7. 10.1177/030098587901600404 452315

[8] Bennett AJ, Paskey AC, Ebinger A, Pfaff F, Priemer G, Höper D, et al. Author Correction: Relatives of rubella virus in diverse mammals. Nature. 2020;588:E2. 10.1038/s41586-020-2897-1 33199919

[9] Bennett AJ, Paskey AC, Ebinger A, Pfaff F, Priemer G, Höper D, et al. Relatives of rubella virus in diverse mammals. Nature. 2020;586:424–8. 10.1038/s41586-020-2812-9 33029010 PMC7572621

[10] de le Roi M, Puff C, Wohlsein P, Pfaff F, Beer M, Baumgärtner W, et al. Rustrela virus as putative cause of nonsuppurative meningoencephalitis in lions. Emerg Infect Dis. 2023;29:1042–5. 10.3201/eid2905.230172 37081716 PMC10124629

[11] Voss A, Schlieben P, Gerst S, Wylezich C, Pfaff F, Langner C, et al. Rustrela virus infection - An emerging neuropathogen of red-necked wallabies (*Macropus rufogriseus*). Transbound Emerg Dis. 2022;69:4016–21. 10.1111/tbed.14708 36135593

[12] Pfaff F, Breithaupt A, Rubbenstroth D, Nippert S, Baumbach C, Gerst S, et al. Revisiting rustrela virus: new cases of encephalitis and a solution to the capsid enigma. Microbiol Spectr. 2022;10:e0010322. 10.1128/spectrum.00103-22 35384712 PMC9045237

[13] Nippert S, Rubbenstroth D, Geers JA, Ebinger A, Hoffmann D, Breithaupt A, et al. Continuous presence of genetically diverse rustrela virus lineages in yellow-necked field mouse reservoir populations in northeastern Germany. Virus Evol. 2023;9:vead048. 10.1093/ve/vead048 PMC10516363 37744713

[14] Armstrong DM, Fitzgerald JP, Meaney CA. Mammals of Colorado. 2nd ed. Boulder (CO): University Press of Colorado; 2011.

[15] Armstrong DM. Rocky Mountain Mammals. 3rd ed. Boulder (CO): University Press of Colorado; 2008.

