# Supplementary figures and images for: Rustrela Virus in Wild Mountain Lion (Puma concolor) with Staggering Disease, Colorado, USA
Source: Emerg Infect Dis. 2024 Aug;30(8):1664–7. doi: 10.3201/eid3008.240411 (PMC11286059; doi:10.3201/eid3008.240411)

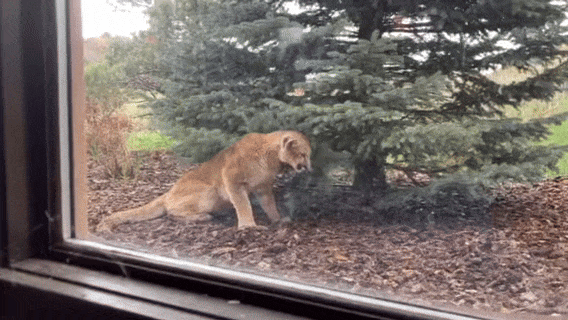

Supplement: Supplementary file 1 [file 24-0411-V.gif]
