# Supplementary material for: Rustrela Virus in Wild Mountain Lion (Puma concolor) with Staggering Disease, Colorado, USA
Source: Emerg Infect Dis. 2024 Aug;30(8):1664–7. doi: 10.3201/eid3008.240411 (PMC11286059; doi:10.3201/eid3008.240411)
Supplement: Appendix — Additional information on detection of rustrela virus in wild mountain lion (Puma concolor) with staggering disease, Colorado, USA. [file 24-0411-Techapp-s1.pdf]

Article DOI: https://doi.org/10.3201/eid3008.240411

EID cannot ensure accessibility for supplementary materials supplied by authors. Readers who have difficulty accessing supplementary content should contact the authors for assistance.

Rustrela Virus in Wild Mountain Lion (*Puma concolor*) with Staggering Disease, Colorado, USA

Appendix

Necropsy and Histopathology

Within three hours after euthanasia, the carcass was transported to a −20°C freezer and then thawed for postmortem examination 2 weeks after freezing. Tissues were preserved in 10% neutral buffered formalin for histologic examination. Tissues collected in formalin included brain, spinal cord, liver, spleen, lung, heart, kidney, stomach, duodenum, pancreas, jejunum, ileum, cecum, colon, ileocecal lymph node, tongue, haired skin, gluteal muscle, adrenal gland, and thyroid gland. Tissues collected and frozen for possible ancillary diagnostics included brain, spinal cord, retropharyngeal lymph node, liver, spleen, adipose tissue, stomach contents, and premolar. Formalin-fixed tissues were embedded in paraffin, sectioned at 5–6 µm, and sections examined by light microscopy after hematoxylin and eosin staining.

Diagnostic Testing for Encephalitic Pathogens

Initial diagnostics for encephalitic pathogens were conducted as shown in Appendix Table 1. All of these tests were performed as standard fee-for-service diagnostics by the Colorado State University Veterinary Diagnostic Laboratory. None of the targeted pathogens were detected by these tests.

Metatranscriptome Analysis

Tissues from pooled brain and spinal cord, targeting gray matter, were submitted for RNA extraction and metatranscriptome sequencing using an Illumina NovaSeq 6000 (2×150 bp)

through Novogene Research Services (Novogene, Beijing, China). The resulting raw sequence reads were initially trimmed using Trim Galore (v0.6.10; (*1*)) and Cutadapt (v 4.0; (*2*)) running in automated adaptor detection and paired-end mode. Trimmed raw reads were then de novo assembled using rnaSPAdes (v3.15.5; (*3*)) and Megahit (v1.2.9; (*4*)). The resulting contigs were then taxonomically classified using DIAMOND blastx (v2.1.8.162; (*5*)) against the non-redundant protein (nr) database from NCBI (version from Apr 2023; (*6*)). Contigs matching potential viral sequences were selected and checked using BLASTn (v2.13.0+; (*7*)) against the non-redundant nucleotide (nt) database from NCBI (version from Apr 2023; (*6*)).

Phylogenetic analysis

For a general taxonomic classification of the novel RusV sequence from a free-ranging mountain lion (Colorado, USA) the predicted amino acid sequence of the structural polyprotein (sPP) was selected for phylogenetic analysis as suggested by the ICTV study group (*8*). The sPP contains the mature peptides of the capsid and the two envelope glycoproteins E2 and E1. The amino acid sequence of sPP was then aligned with appropriate references from other rubiviruses or currently unclassified but related matonavirids from GenBank using MUSCLE (v3.8.425; (*9*)). The tree was constructed using IQ-TREE (version 2.2.3; (*10*)) using optimal model selection (*11*) and statistical support with 100.000 replicates each for ultrafast bootstrap (*12*) and SH-aLRT test.

For a more detailed analysis, the whole genome sequences of all available RusV entries available from GenBank (25.01.2024) were aligned along with the novel sequence using MAFFT (version 7.490; (*13*)). A phylogenetic tree was calculated as described above. The alignment was also used to calculate the mean genetic distance (K80 model) between the novel RusV sequence and the RusV sequences from Germany and Sweden/Austria using a sliding window (window: 400 nt, step size: 50 nt).

Nucleotide and amino acid identities were calculated between all pairs of available RusV whole genome sequences, and non-structural polyprotein (nsPP) and sPP, respectively. Mean pairwise identities were then calculated for RusV sequences from Germany and Sweden/Austria and compared to the novel RusV from USA.

PCR

Previously developed real-time reverse transcription PCR (RT-PCR) primers and probe (*14*) targeting the 5′ terminus of RusV were adapted for consensus sequence homology to the Colorado, USA mountain lion sequence and the European RusV sequences, as given in Appendix Table 2. RNA was extracted from a pooled sample of the suspect mountain lion brain and spinal cord, targeting gray matter, using an Allprep Power Viral DNA/RNA kit (Qiagen, Germantown, MD) and DNase Max kit (Qiagen) according to manufacturer's instructions. We used a TaqMan Fast Virus 1-Step Master Mix (Thermo Fisher Scientific, Waltham, MA), and VetMAX Xeno Internal Positive Control-VIC Assay with VetMAX Xeno Internal Positive Control RNA (Thermo Fisher Scientific) according to manufacturer's instructions. We used 5.0 μL of extracted template RNA in a total reaction volume of 25 μL. Reactions were cycled using an Eco real-time PCR thermocycler (Illumina, San Diego, CA) and Eco software (Illumina, v. 4.0.07.0) for analysis. Automated thresholds were used due to lack of known positive control material for standard curve dilutions in Colorado, USA. Negative controls (normal mountain lion pooled brain and spinal cord, and no template control) and internal positive RNA controls were adequate for interpretation.

In situ Hybridization

In situ hybridization for the detection of RusV RNA in brain and spinal cord tissue sections was performed with the RNAScope 2–5 HD Reagent Kit-Red (Advanced Cell Diagnostics) according to the manufacturer's instructions. Probes were custom-designed for the non-structural protein gene of the sequence obtained from the puma brain (Catalog number 1323611-C1). As technical assay controls, a positive control probe (*Felis catus* peptidylprolyl isomerase B, *PPIB*) and a negative control probe (dihydrodipicolinate reductase) were included. Archived brain tissue slides from a snow leopard (*Panthera uncia*) diagnosed with ocular, bilateral anterior segment dysgenesis were included as negative controls. All slides were scanned using a Hamamatsu S60 scanner, evaluation was done using the NDPview.2 plus software (Version 2.8.24, Hamamatsu Photonics, K.K. Japan). Interpretation was performed by a board-certified pathologist (DiplECVP). Representative photographs of control slides are shown in Appendix Figure 4.

References

1. Krueger F. 2023. Trim Galore. https://www.bioinformatics.babraham.ac.uk/projects/trim_galore

2. Martin M. Cutadapt removes adapter sequences from high-throughput sequencing reads. EMBnet J. 2011;17:10. https://doi.org/10.14806/ej.17.1.200

3. Bushmanova E, Antipov D, Lapidus A, Prjibelski AD. rnaSPAdes: a de novo transcriptome assembler and its application to RNA-Seq data. Gigascience. 2019;8:8. PubMed https://doi.org/10.1093/gigascience/giz100

4. Li D, Liu C-M, Luo R, Sadakane K, Lam T-W. MEGAHIT: an ultra-fast single-node solution for large and complex metagenomics assembly via succinct de Bruijn graph. Bioinformatics. 2015;31:1674–6. PubMed https://doi.org/10.1093/bioinformatics/btv033

5. Buchfink B, Reuter K, Drost H-G. Sensitive protein alignments at tree-of-life scale using DIAMOND. Nat Methods. 2021;18:366–8. PubMed https://doi.org/10.1038/s41592-021-01101-x

6. Sayers EW, Bolton EE, Brister JR, Canese K, Chan J, Comeau DC, et al. Database resources of the national center for biotechnology information. Nucleic Acids Res. 2022;50(D1):D20–6. PubMed https://doi.org/10.1093/nar/gkab1112

7. Camacho C, Coulouris G, Avagyan V, Ma N, Papadopoulos J, Bealer K, et al. BLAST+: architecture and applications. BMC Bioinformatics. 2009;10:421. PubMed https://doi.org/10.1186/1471-2105-10-421

8. Mankertz A, Chen M-H, Goldberg TL, Hübschen JM, Pfaff F, Ulrich RG; Ictv Report Consortium. ICTV Virus Taxonomy Profile: *Matonaviridae* 2022. J Gen Virol. 2022;103:103. PubMed https://doi.org/10.1099/jgv.0.001817

9. Edgar RC. MUSCLE: multiple sequence alignment with high accuracy and high throughput. Nucleic Acids Res. 2004;32:1792–7. PubMed https://doi.org/10.1093/nar/gkh340

10. Minh BQ, Schmidt HA, Chernomor O, Schrempf D, Woodhams MD, von Haeseler A, et al. IQ-TREE 2: new models and efficient methods for phylogenetic inference in the genomic era. Mol Biol Evol. 2020;37:1530–4. PubMed https://doi.org/10.1093/molbev/msaa015

11. Kalyaanamoorthy S, Minh BQ, Wong TKF, von Haeseler A, Jermiin LS. ModelFinder: fast model selection for accurate phylogenetic estimates. Nat Methods. 2017;14:587–9. PubMed https://doi.org/10.1038/nmeth.4285

12. Hoang DT, Chernomor O, von Haeseler A, Minh BQ, Vinh LS. UFBoot2: improving the ultrafast bootstrap approximation. Mol Biol Evol. 2018;35:518–22. PubMed https://doi.org/10.1093/molbev/msx281

13. Katoh K, Misawa K, Kuma K, Miyata T. MAFFT: a novel method for rapid multiple sequence alignment based on fast Fourier transform. Nucleic Acids Res. 2002;30:3059–66. PubMed https://doi.org/10.1093/nar/gkf436

14. Matiasek K, Pfaff F, Weissenböck H, Wylezich C, Kolodziejek J, Tengstrand S, et al. Mystery of fatal ‘staggering disease’ unravelled: novel rustrela virus causes severe meningoencephalomyelitis in domestic cats. Nat Commun. 2023;14:624. PubMed https://doi.org/10.1038/s41467-023-36204-w

Appendix Table 1. Initial diagnostics performed to investigate differential diagnoses for meningoecephalomyelitis in a free-ranging mountain lion*

Targeted pathogen	Test	Tissue	Result
Feline panleukopenia virus	Real-time RT-PCR	Brain	Not detected
Canine distemper virus	Real-time RT-PCR	Brain	Not detected
West Nile virus	Real-time RT-PCR	Brain	Not detected
Toxoplasma gondii	PCR	Brain	Not detected
Influenza A virus	Real-time RT-PCR	Spinal cord	Not detected
Rabies virus	Immunofluorescence	Brainstem	Not detected
Feline coronavirus (feline infectious peritonitis)	Immunohistochemistry	Brain (cerebral cortex)	Not detected

*RT-PCR, reverse transcription PCR.

Appendix Table 2. Real-time RT-PCR assay details, with primers and probe adapted for consensus homology to the Colorado, USA mountain lion RusV sequence and European sequences*

Primer/Probe/Condition	Details
RusV_234 (fw) adapted primer	CCCYGTGTTCCTAGGCAC; 0.8 µM
RusV_323 (rv) adapted primer	TCGCCCCATTCDACCCAATT; 0.8 µM
RusV_256 adapted probe	GTGMGCGACCACCCAGCACTCCA; 0.4 µM
Probe modifications	5′FAM/ZEN/3′IBFQ
VetMAX Xeno IPC RNA	2 µL added to samples before lysis
VetMAX Xeno IPC-VIC assay	1 µL added to 25 µL PCR reaction mix
Cycling conditions	45°C × 10 min
	95°C × 10 min
	45 cycles of:
	95°C × 15 s
	60°C × 30 s
	72°C × 30 s

*Adapted from (*14*).

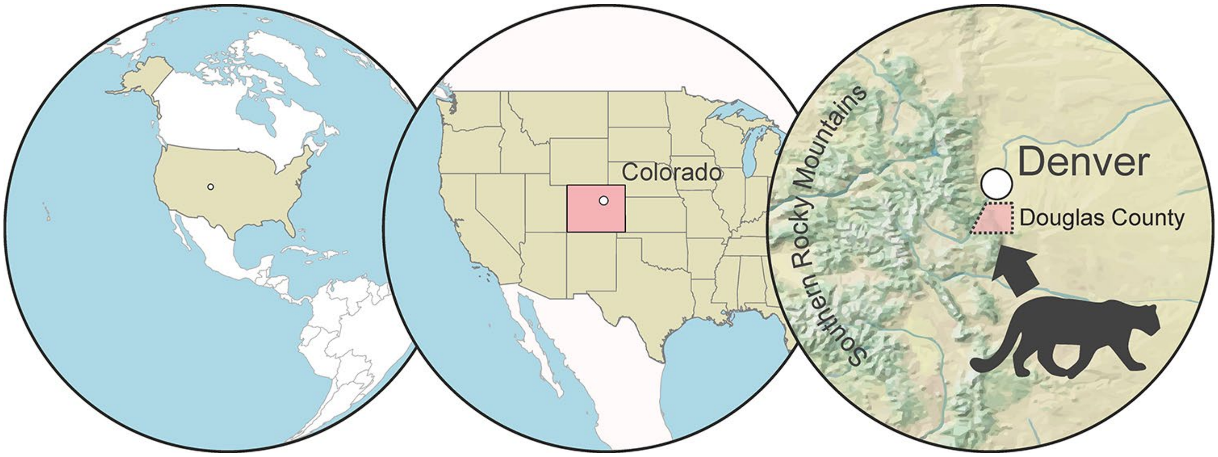

Appendix Figure 1. The free-ranging mountain lion (*Puma concolor*) was found in a residential area of Douglas County which is located south of Denver (Colorado, USA). Made with Natural Earth.

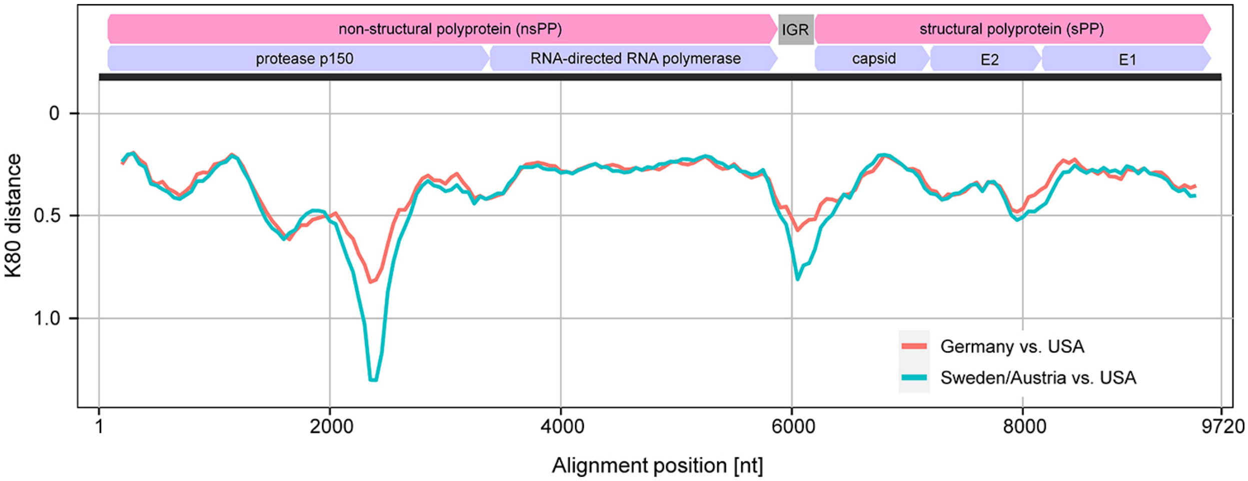

Appendix Figure 2. The mean genetic distance (K80 model) between the novel RusV sequence from a free-ranging mountain lion from Colorado, USA, and the published RusV sequences from Germany or Sweden/Austria was calculated in a sliding window (window: 400 nt, step size: 50 nt) based on a whole genome nucleotide alignment (9,720 nt). The genomic architecture of RusV (deduced from MN552442.2) is shown: pink arrows represent ORFs that encode the polyproteins nsPP and sPP, while blue arrows represent mature peptides that are produced by cleavage from the polyproteins. The intergenic region (IGR) is located between both nsPP and sPP.

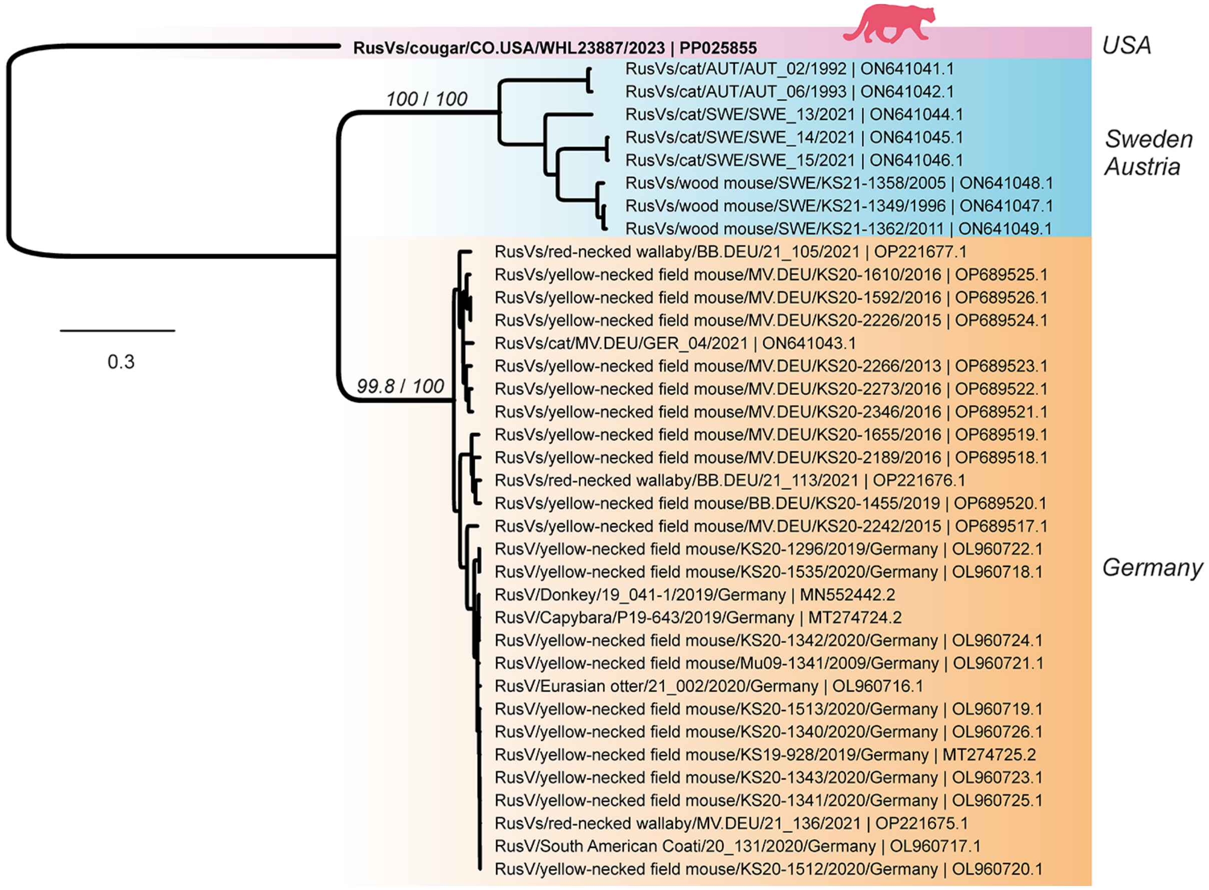

Appendix Figure 3. Phylogenetic position of rustrela virus (RusV) based on a nucleotide alignment of whole genomes (9,720 nt). The novel RusV sequence from a free-ranging mountain lion from Colorado, USA is highlighted using bold text. RusV genetic groups are highlighted and labeled according to their geographic origin. Alignment was done using MAFFT (version 7.490; (*13*)). The tree was constructed using IQ-TREE (version 2.2.3; (*10*)) using optimal model selection and statistical support with 100.000 replicates each for ultrafast bootstrap (*12*) and SH-aLRT test. Statistical support is shown for main branches using the format [ultrafast bootstrap/SH-aLRT]. Scale bar indicates nucleotide substitutions per site.

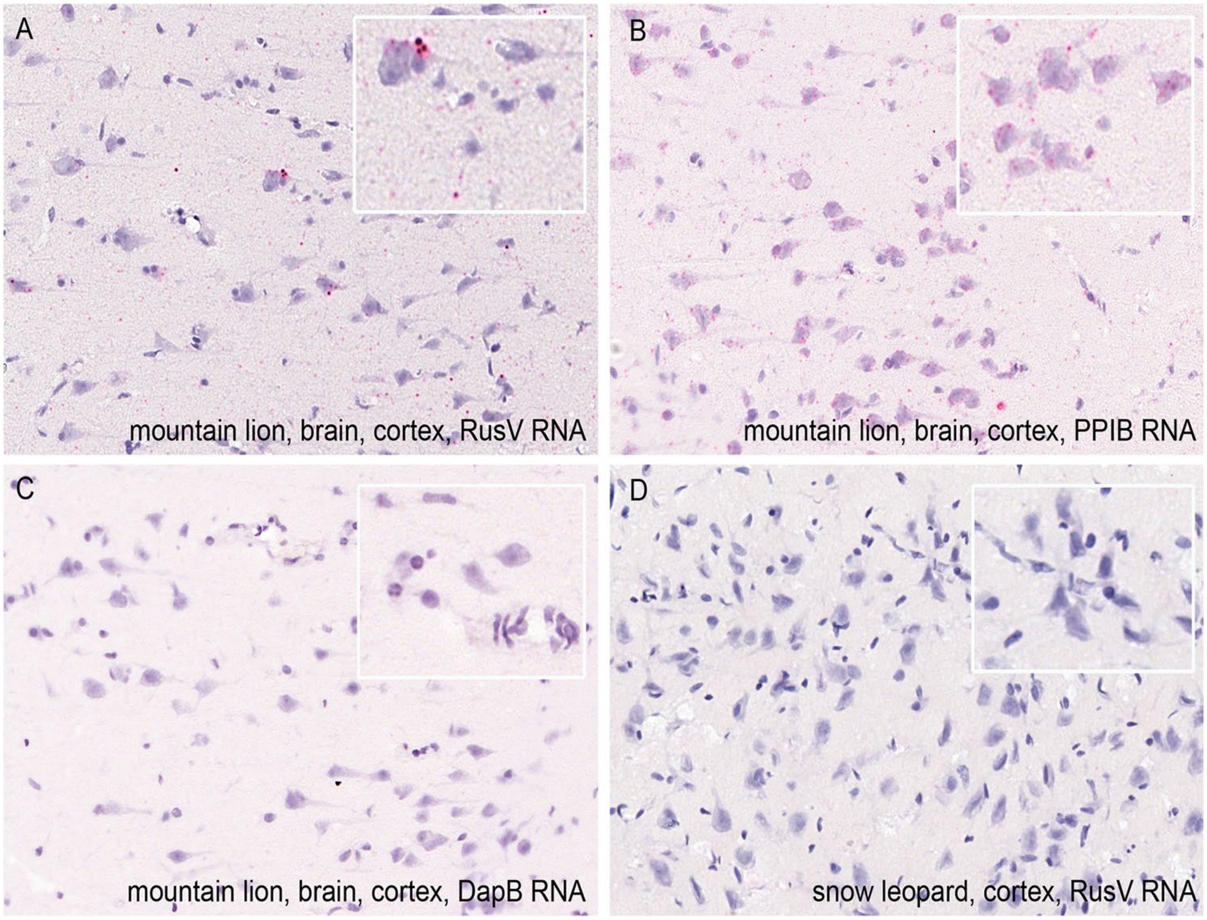

Appendix Figure 4. RNA in situ hybridization for rustrela virus (RusV) RNA detection in brain tissues including controls. A) Chromogenic labeling (fast red) with probes against the NSP-coding region of RusV, visible in in cortical neurons and in the neuropil of the mountain lion. B) Technical, positive control probe (*Felis catus*) peptidylprolyl isomerase B, *PPIB* yielding disseminated labeling. C) Technical negative control probe (dihydrodipicolinate reductase, *DapB*) showing no labeling. D) Archived brain tissue slides from a snow leopard (*Panthera uncia*) tested negative for RusV-RNA. All slides counterstained with Mayer's hematoxylin counterstain, inlays show higher magnifications.
